# Biomolecular Clusters Distribution up to Mega Dalton Region Using MALDI-Quadrupole Ion Trap Mass Spectrometer

**DOI:** 10.3390/ijms19092789

**Published:** 2018-09-17

**Authors:** Yung-Kun Chuang, Szu-Hsueh Lai, Jung-Lee Lin, Chung-Hsuan Chen

**Affiliations:** 1 Master Program in Food Safety, Taipei Medical University, 250 Wusing Street, Taipei 11031, Taiwan; ykchuang@tmu.edu.tw; 2 Genomics Research Center, Academia Sinica, 128 Academia Road, Section 2, Taipei 11529, Taiwan; szuhsuehlai@yahoo.com (S.-H.L.); harrylin@gate.sinica.edu.tw (J.-L.L.); 3 Institute of Atomic and Molecular Sciences, Academia Sinica, No. 1, Section 4, Roosevelt Road, Taipei 10617, Taiwan; 4 Department of Chemistry, National Taiwan University, No. 1, Section 4, Roosevelt Road, Taipei 10617, Taiwan; 5 Department of Bio-Industrial Mechatronics Engineering, National Taiwan University, No. 1, Section 4, Roosevelt Road, Taipei 10617, Taiwan

**Keywords:** MALDI, cytochrome c, bovine serum albumin (BSA), large clusters, quantitative distribution

## Abstract

We present the first report on complete cluster distributions of cytochrome c (molecular weight of 12.4 kDa) and bovine serum albumin ((BSA), molecular weight of 66.4 kDa) with mass-to-charge ratio (*m*/*z*) reaching 350,000 and 1,400,000, respectively, by matrix-assisted laser desorption/ionization (MALDI). Large cluster distributions of the analytes were measured by our homemade frequency-scanned quadrupole ion trap (QIT) mass spectrometer with a charge detector. To our knowledge, we report the highest *m*/*z* clusters of these two biomolecules. The quantitative results indicate that large clusters ions of cytochrome c and BSA follow the power law (*r*^2^ > 0.99) with cluster size distribution, which provides experimental evidence for the laser ablation studies of MALDI.

## 1. Introduction

**Figure 1:**
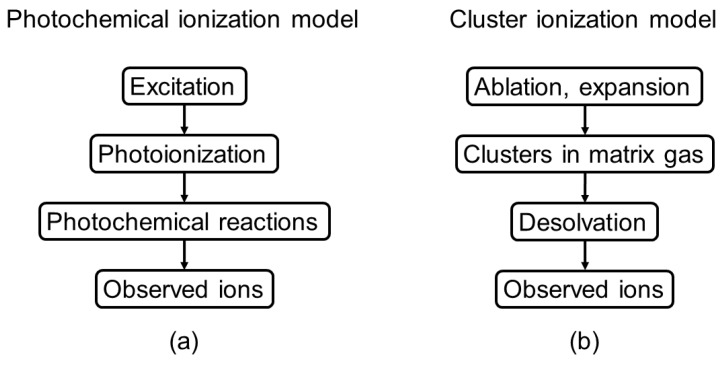
(**a**) Schematic diagram of photoionization and subsequent reaction pathways in matrix-assisted laser desorption/ionization mass spectrometry (MALDI)-MS, as proposed by Ehring, Karas, and Hillenkamp in 1992 (see the literature [8] for further details). (**b**) Sketch of the major processes proposed in the cluster models of MALDI ionization, as proposed and largely developed by the Karas group (see the literature [22] for further details).

Matrix-assisted laser desorption/ionization (MALDI), an important and valuable ionization technique used in mass spectrometry [1,2], has been widely applied for the analysis of large biomolecules such as proteins and peptides [3]. MALDI has the advantages of producing high mass ions so that the mass spectra are more suitable for biomolecule analysis [4]. In recent years, MALDI has been successfully demonstrated to contribute to biomedical [5], chemical [6], and pharmaceutical [7] research, which shows the impressive progress in diversified applications. Researchers found that understanding the MALDI mechanism is critically important when conducting qualitative and quantitative measurements, which is related to the reproducibility and mass resolution of experiments. To date, photochemical ionization [8,9,10,11,12,13,14,15,16] and cluster ionization [17,18,19,20,21,22,23] are the two major hypotheses to explain many of the experimental results in MALDI. The schematic diagrams of these two hypotheses are shown in Figure 1a,b. For the photochemical ionization model, the ions of the analyte are considered to be produced from a protonation/deprotonation process involving an analyte molecule colliding with a matrix ion in the gas phase. Energy pooling and multiphoton absorption are the major processes leading to the photoionization of a matrix molecule. All of the analyte ions have to come from collision processes between neutral analyte molecules and protonated or deprotonated matrix molecules. The cluster ionization model assumes that the protonated analyte ions are from hot matrix clusters that are produced during laser ablation. Analyte ions are produced in the gas phase by the desolvation of neutral matrix molecules. Nevertheless, it seems to be difficult to explain the predominant production of mono-charged ions for biomolecules with a broad mass range, as electrospray ionization (ESI) produces mostly multiply charged ions for large biomolecules. Even though numerous studies aimed to provide possible explanations for ion formation in MALDI [8,9,10,11,12,13,14,15,16,17,18,19,20,21,22,23,24,25,26,27,28,29,30,31,32,33,34,35,36,37,38,39], many experimental observations such as sweet spots and the matrix suppression effect still cannot be fully interpreted by these hypotheses.

On the other hand, it is equally noteworthy that a hypothesis of molecular dynamics (MD) was proposed through the simulation of laser ablation of organic targets to study the MALDI process [40,41,42,43,44,45,46,47,48,49]. The MD model can be used to explain the differences between the ablative photodecomposition and thermal processes in organic polymers. A comprehensive investigation for cluster productions has been discussed through a series of large-scale molecular dynamics simulations of laser ablation [44]. The report summarizes that condensation is used to explain the observation of small clusters, while the direct ejection of clusters from the target is more likely to be responsible for the production of larger clusters. The surface region, overheated up to the limit of its thermodynamic stability by short-pulse laser irradiation, is predicted to undergo a rapid transition from an overheated liquid to a mixture of vapor and liquid droplets [50,51,52,53,54]. Large droplets are ejected as a result of a transient melting and the motion of a liquid, caused by steep thermal gradients and a relaxation of the laser induced pressure. The ejection of large liquid droplets and/or solid particulates can be observed in the regime of stress confinement by the relaxation of the laser-induced stresses. A power–law dependence was then proposed for cluster size distribution as *Y*(*N*) ∝ *N*^−*τ*^, where *N* is the clusters’ size and *τ* represents the power–law exponent. Previous studies have measured the polymer cluster distribution in a micrometer-size region by scanning electron microscope imaging [55]. The relationship between cluster distribution of organic acid and the model of molecular dynamics has also been demonstrated in evaluating the entire size distribution of sinapinic acid (SA) clusters in the mass region from hundreds to mega Dalton by MALDI [56]. Until presently, however, no literature exists for the entire size distribution of large biomolecular clusters from monomer to the mega Dalton region. There is a strong need to obtain experimental data from the measurements of biomolecules, as the observed entire size distributions might contain critical information about the mechanism for clusters to be produced, and its relationship to MALDI mechanism. Therefore, the objective of the current study is to examine the distributions of large biomolecular clusters in terms of quantitative approach, to provide experimental evidence for cluster productions during the MALDI process.

In the past, many works on MALDI were pursued with time-of-flight (TOF) mass spectrometers. A commercially available mass spectrometer is normally equipped with an electro multiplier, a Channeltron, or a microchannel plate (MCP) as a detecting device. The detection efficiency of these charge amplification detectors involves secondary electron ejection efficiency, which is strongly related to the velocity of the ions. As the velocity of an ion is inversely proportional to the root square of the mass at a fixed energy, the detection sensitivity is very poor when the molecular weight is above 100,000 Da. For very heavy biomolecular ions, the detection efficiency decreases when mass-to-charge ratio (*m*/*z*) increases [57]. Therefore, most commercial MALDI-TOF mass spectrometers are not suitable for detecting very large biomolecular clusters due to the extremely low detection efficiencies for ions with *m*/*z* higher than 200,000. The signal intensity distribution of the biomolecular clusters also does not represent the real distribution of the ions. In order to avoid this bias, two types of detectors, cryogenic and inductive, have been developed to overcome the detection limitation of ions with an extremely large *m*/*z* [58,59]. The sensitivity of a cryogenic detector is independent of the mass. Cryogenic detectors have been demonstrated to detect very large molecules, such as immunoglobulin M (IgM) and von Willebrand factor proteins [60]. However, the effective area of a typical cryogenic detector is much smaller than that of a typical MCP. Therefore, the overall detection efficiency is lower. The other high-mass particle detector is a specific charge detector for MALDI-TOF [61]. We used a charge detector to replace an electron amplification device in our homemade frequency-scanned quadrupole ion trap (QIT) mass spectrometer [56,62]. A charge detector can only detect the charge number ions. It is not a concern at all for the mass of ions. Therefore, a broad signal intensity distribution of biomolecular clusters can be quantitatively measured by our instrument, as the detection efficiency in a broad mass region is fairly equal. It can truly represent the real cluster distribution.

In the present study, our instrument [56,62] was used to measure a broad mass region of biomolecular cluster ions with different sizes for MALDI. Two well-known biomolecules with a medium molecular weight, cytochrome c and bovine serum albumin (BSA), were employed to obtain quantitative distribution of cluster size distributions with mass-to-charge ratio (*m*/*z*) reaching 350,000 and 1,400,000, respectively. The reasons for choosing cytochrome c and BSA as analytes were as follows: (1) more sufficient amount of cluster sizes (e.g., 28-mer cytochrome c and 21-mer BSA) can be observed in the mass region, and (2) these proteins are more suitable for evaluating the entire size distribution and variation trend of biomolecular clusters instead of using larger proteins such as immunoglobulin G (IgG), immunoglobulin A (IgA), and IgM. In the current study, we have demonstrated that our homemade frequency-scanned QIT mass spectrometer with a charge detector can be used to quantitatively detect very large cluster ions. The experimental results also demonstrated the existences of very large bio-clusters in MALDI. In order to prove that the sequential peaks in mass spectra are mainly from protein clusters, a commercial MALDI time-of-flight mass spectrometer (Bruker, Ultraflex II TOF/TOF) was also used to obtain the cluster spectra of cytochrome c at a lower mass region (10,000–100,000 Da). As the quantitative measurements of large cluster ions are achieved, the entire size distributions of cytochrome c and BSA clusters can provide information on the currently proposed hypotheses of cluster productions for the MALDI process.

## 2. Results and Discussion

### 2.1. Large Clusters of the Analytes

**Figure 2:**
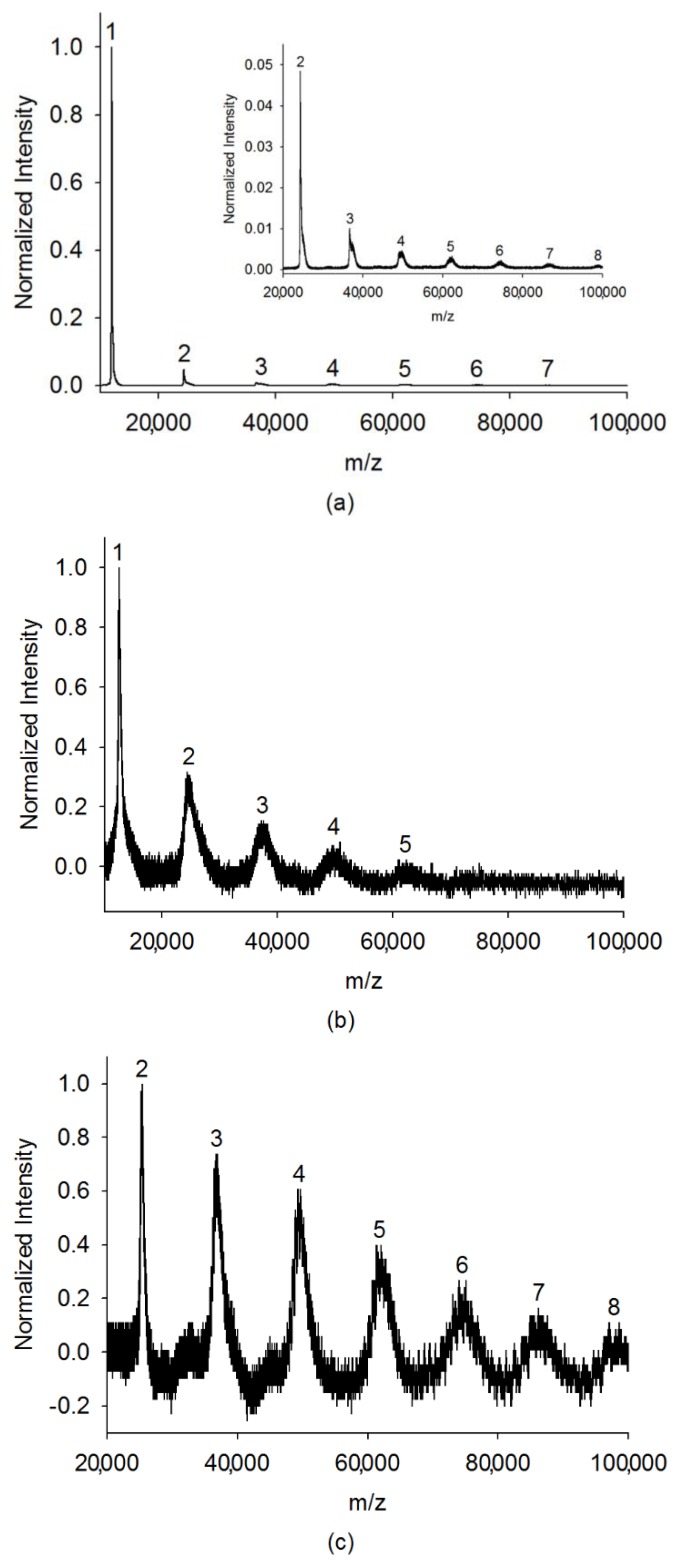
(**a**) Cytochrome c clusters with *m*/*z* from 10,000 to 100,000 Da obtained by the Bruker Ultraflex II MALDI-time-of-flight (TOF)/TOF mass spectrometer. The inside box is the magnified mass spectrum for a molecular weight from 20,000 to 100,000 Da. (**b**) Cytochrome c clusters with *m*/*z* from 10,000 to 100,000 Da and (**c**) from 20,000 to 100,000 Da obtained by our homemade frequency-scanned quadrupole ion trap (QIT) mass spectrometer with a charge detector.

Figure 2a shows the mass spectrum of cytochrome c for a low cluster region obtained by the Bruker Ultraflex II MALDI-TOF/TOF mass spectrometer, and the insertion box shows a magnifying spectrum of the mass range from 20,000 to 100,000 Da. The clusters’ peaks from monomer to heptamer can be clearly seen in this region. The consistent observation results of cytochrome c for low cluster regions were also obtained by our instrument, as shown in Figure 2b,c. Frequency scanning was performed from 70 kHz to 10 kHz and from 50 kHz to 10 kHz with RF voltage at 800 Vpp, respectively. If the peaks are mainly from protein-matrix cluster, the smearing of the mass spectrum on the high mass side should be observed. In addition, only equal spaces between two neighboring peaks with the gap of *m*/*z* of 12,384 (molecular weight of cytochrome c monomer) were observed. This observation clearly indicates that these peaks mainly represent protein clusters, even though a small number of matrix adducts may also contribute to the ion signal.

**Figure 3:**
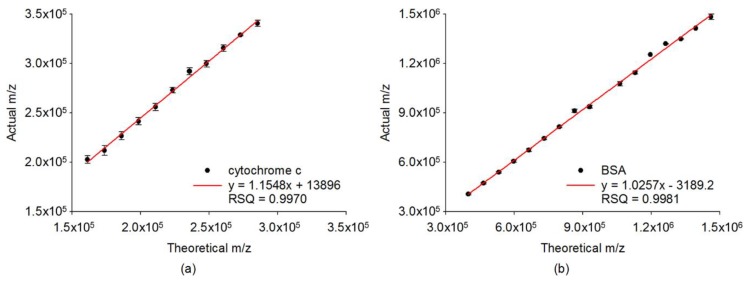
Mass calibration curves of expected *m*/*z* (theoretical value) versus measured *m*/*z* (actual value) for mass spectra of (**a**) cytochrome c with mass range from 150 kDa to 350 kDa, and (**b**) bovine serum albumin (BSA) with a mass range from 300 kDa to 1500 kDa.

Several preliminary examinations for cytochrome c and BSA were conducted to determine the optimal parameters for measurement by using our instrument. Different helium pressures inside the ion trap were applied for two different biomolecules. The optimal helium pressures for trapping the positive ions of cytochrome c and BSA were about 50 mTorr and 65 mTorr, respectively. In order to effectively improve the signal-to-noise (*S*/*N*) ratio of large clusters observed in the mass spectra, the accumulation of enough ions before detection was employed for all of the experiments. A stepwise frequency scan was performed by setting a proper start frequency corresponding to the molecular weight of the targeted analyte cluster so as to identify each size of the clusters. With different molecular weights, the trapping frequency was swept using step-scan for various mass ranges [56,62]. The measured *m*/*z* (actual value) were larger than the expected *m*/*z* (theoretical value), because of the time delay between the ejected frequency and flight time of ions from the ion trap to charge detector. Therefore, the calibration curves for various mass ranges were built to correct the *m*/*z* of all of the mass spectra. As shown in Figure 3, a calibration curve of expected *m*/*z* versus measured *m*/*z*, with a mass range from 150 kDa to 350 kDa was attained for cytochrome c with RF voltage at 1200 Vpp. The coefficient of determination (*r*^2^) was determined as 0.9970. For BSA, a calibration curve was acquired with the mass spectra ranging from 300 kDa to 1500 kDa, and the mass accuracy was 21,403 ppm at a RF voltage of 1600 Vpp, with *r*^2^ being 0.9981. The measured *m*/*z* of each size of cluster shown in the mass calibration curves was the average value of more than 50 data points in order to assure mass accuracy. The mass calibration curves with very a high correlation for the low-mass regions were also built to completely cover the small cluster distributions of cytochrome c and BSA, respectively. These calibration curves confirm the mass accuracy, ranging from a medium to high mass region. The corrected *m*/*z* for the peaks in the mass spectra thus can be calculated according to the corresponding linear regression equations.

**Figure 4:**
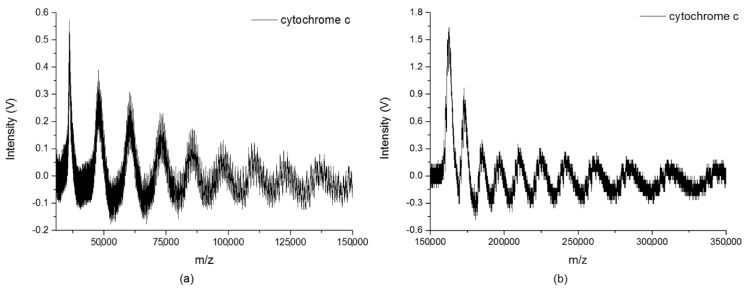
(**a**) Cytochrome c clusters in low-mass region. (**b**) Cytochrome c clusters with *m*/*z* from 150 kDa to 350 kDa.

**Figure 5:**
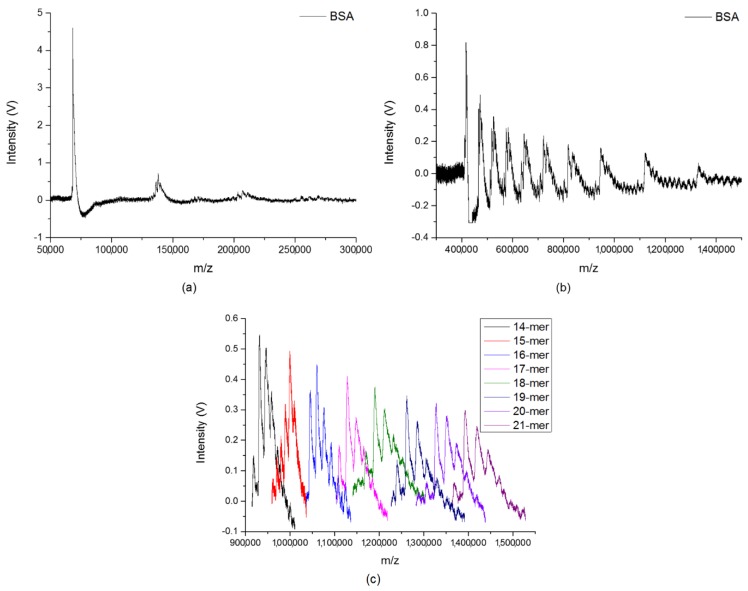
(**a**) The BSA clusters in low-mass region. (**b**) BSA clusters with *m*/*z* from 300 kDa to 1500 kDa. (**c**) Signals of specific BSA cluster with various sizes (14-mer to 21-mer) in very high mass region under eight different ranges of scanning frequencies, respectively. The gap between each size of BSA clusters is around 66 kDa.

A broad mass range from 10 kDa to 1500 kDa has been demonstrated by our instrument. The mass spectra in the low-mass region were first measured to completely cover the small cluster distributions of cytochrome c and BSA, as shown in Figure 4a and Figure 5a, respectively. Figure 4b shows cytochrome c clusters from 150 kDa to 350 kDa after mass calibration. The helium pressure inside the ion trap was maintained at 50 mTorr in order to increase the trapping efficiency. The spectrum was obtained with the accumulation of ions for 100 laser shots, and the data were collected with a scan rate of 5 × 10^5^ Hz/s. The asymmetric shapes of the clusters’ peaks observed in the figure are due to the electronic characteristic of our charge detector. For a single peak, the left side to the pulse height of the peak is the charge collection time and the other side is the discharge time, and the discharge time is much longer than the charge collection time. When detecting very heavy biomolecular ions, a small number of ions may reach the charge detector during the discharging period, as their flight time is longer than the charge collection time. This phenomenon may lead to the skewness at the back side of the peaks, and reduce the mass resolution in the high-mass region. Nevertheless, it is unlikely to affect the mass calibration, because the number of involved ions is small. The clusters’ peaks of cytochrome c, shown in the figure, can be clearly seen in the mass range up to 350 kDa. This observation confirmed the existence of large clusters of cytochrome c during the MALDI process.

The BSA clusters with the correct reading of the corresponding mass from 300 kDa to 1500 kDa after mass calibration are shown in Figure 5b. Frequency scanning was performed from 17 kHz to 7 kHz, with a RF voltage at 1600 Vpp. The helium pressure inside the ion trap was maintained at 65 mTorr during the experiment, so as to reduce the kinetic energy from the high molecular weight. The spectrum was obtained with the accumulation of ions for 200 laser shots, and the data were collected with a scan rate of 5 × 10^5^ Hz/s. Even if the mass resolution of the observed clusters reduced in the very high-mass region, the peaks for various BSA clusters with *m*/*z* reaching to 1400 kDa were clearly seen in the figure. To obtain the correct *m*/*z* values of each size of BSA clusters, it was required to narrow down the mass range of observation by setting a proper range of scanning frequencies for each measurement. Figure 5c shows the signals of the specific BSA clusters with various sizes (14-mer to 21-mer) in a very high mass region, by setting eight different ranges of scanning frequencies, respectively. The gap between each size of BSA clusters is around 66 kDa. For large biomolecules with different molecular weights, Figure 4 and Figure 5 demonstrated the real cluster distributions in MALDI.

### 2.2. Quantitative Analysis on Cluster Distributions of the Analytes

**Figure 6:**
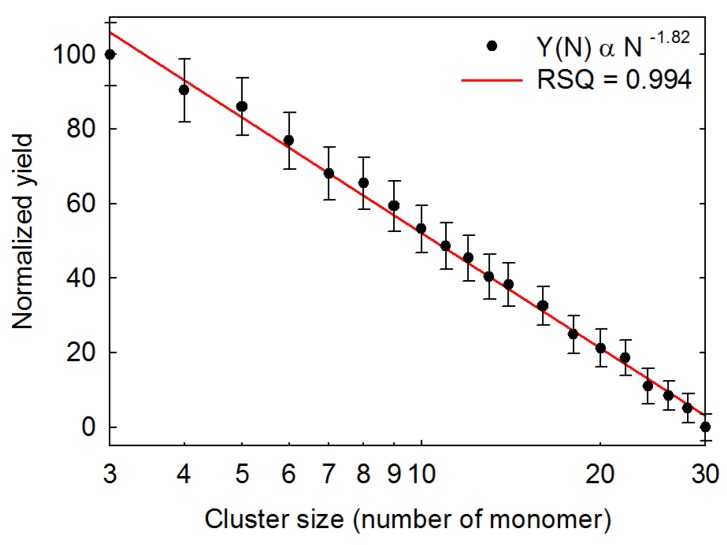
Cluster size distribution of cytochrome c from trimer to 30-mer. The cluster size (*x*-axis) and the normalized relative intensity (*y*-axis) are plotted on a base-10 logarithmic scale.

To quantitatively measure a broad distribution of biomolecular clusters by our instrument, the ions from MALDI were accumulated in the ion trap with 10 laser shots. The number of laser shots was fixed somewhat low here to avoid signal saturation by small cluster ions. The trapping frequency was swept to the step-scan various mass ranges. As stepwise scanning is not a continuous detection in the mass range, only the relative intensity distributions were recorded. The plot of the relative intensity distribution of cytochrome c from trimer to 30-mer is shown in Figure 6, with laser fluence around 9 mJ/mm^2^. The cluster size (*x*-axis) and the normalized relative intensity (y-axis) were plotted on a base-10 logarithmic scale. The relative intensity of each size of cluster shown in the figure is the average value of more than 50 data points, so as to achieve a more objective evaluation of the signal intensity. The distribution and variation trend of the experimental data can be readily observed. The higher the mass cluster of cytochrome c, the lower the relative intensity. A power–law dependence relationship with *r*^2^ being 0.994 fits very well for the quantitative description of the cluster size distribution. A *τ* value of 1.82 for the power–law exponent was obtained according to the Equation (1) after the curve fitting.

*Y*(*N*) = 1029*N*^−*τ*^ (1)

**Figure 7:**
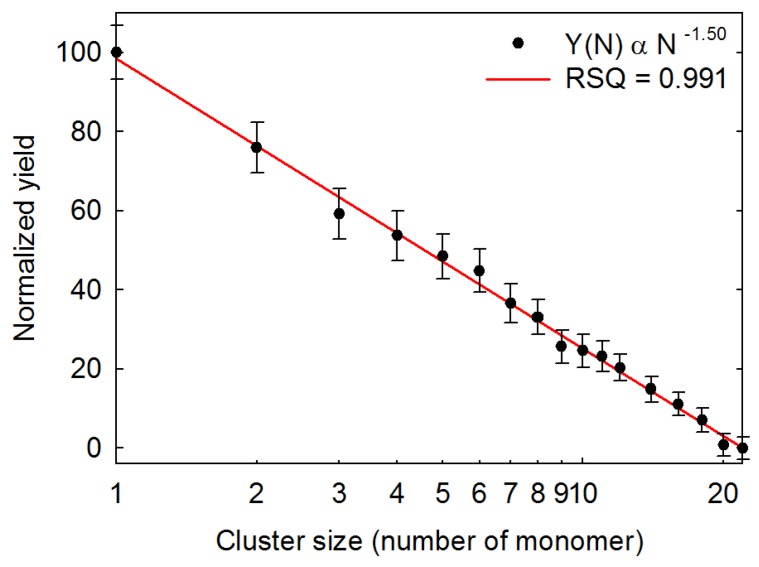
Cluster size distribution of BSA from monomer to 22-mer. The cluster size (*x*-axis) and the normalized relative intensity (*y*-axis) are plotted on a base-10 logarithmic scale.

Large cluster ions with a size distribution of BSA were also measured by our instrument. The laser fluence was around 9 mJ/mm^2^ and the ions were accumulated in the ion trap with 10 laser shots, in order to avoid signal saturation by the monomer ions. The relative intensity of each size of BSA cluster was the average value of more than 50 data points. As shown in Figure 7, the power-law dependence relationship with *r*^2^ at 0.991 fit very well for the cluster size distribution of BSA from monomer to 22-mer. A *τ* value of 1.50 for the power–law exponent was obtained according to Equation (2) by curve fitting. The cluster size distributions of the two biomolecules are about the same. The decay of the BSA clusters is slower than that of cytochrome c, which is consistent with the molecular dynamics simulations of MALDI in the high-mass region of the distributions [44]. The consistent experimental results for measuring cytochrome c and BSA indicate that large cluster ions of biomolecules from MALDI certainly agree to the power–law cluster size distribution.

*Y*(*N*) = 92*N*^−*τ*^ (2)

Compared to the previous literature [8,9,10,11,12,13,14,15,16,17,18,19,20,21,22,23,24,25,26,27,28,29,30,31,32,33,34,35,36,37,38,39,40,41,42,43,44,45,46,47,48,49,55,56,63,64,65], we present the first report on the evaluation of large biomolecular ion cluster distributions from the monomer to mega Dalton region using the MALDI ion trap mass spectrometer with a charge detector. The first observation of the largest cluster ions from cytochrome c and BSA in this work not only confirm the existences of very large bio-clusters in MALDI, but also shine a light on some of the currently proposed hypotheses. As indicated in Figure 7, the decrease of the two orders in the relative intensity was observed from the monomer ions to the 22-mer ions. The production of such a large amount of large cluster ions by stepwise collisions in the plume seems unlikely, especially at the molar ratios of matrix to analyte of 5000. The cluster size distributions of cytochrome c and BSA indicate that large cluster ions in the high-mass region seem to not be initiated from mono-analyte ion-biomolecule reactions. In addition, bovine insulin 20-mer ions in a MALDI experiment have been reported before [66]. The observation of a large number of polymer ions indicates that the photochemical ionization model may not be the primary process to produce high analyte polymer ions. For the cluster ionization model, it is also difficult to fully support the explanation for ionization of large biomolecules, because of the very low population of large matrix clusters in the high-mass region [56]. Neither the photochemical ionization nor cluster ionization can fully explain the predominant production of large cluster ions for large biomolecules with a broad mass region. A number of scenarios of cluster formation in laser ablation introduced in previous literature [44] seem more likely to interpret the production of a large amount of large biomolecular cluster ions. For matrix–analyte mixtures, the matrix plays a role in the preionization of the analyte during the recrystallization process during the sample preparation [37]. Phase explosion is predicted to occur while the surface region of the sample overheated up to the limit of its thermodynamic stability by the irradiation of a short-pulsed laser. Small clusters with different sizes are formed through zone-melting recrystallization [67], and then large droplets are ejected during the process of hydrodynamic sputtering. According to the experimental results, the power–law dependence relationships seem to be more agreeable to the molecular dynamics simulations of MALDI for biomolecular ion clusters [40,41,42,43,44,45,46,47,48,49].

## 3. Materials and Methods

### 3.1. Instrumentation

**Figure 8:**
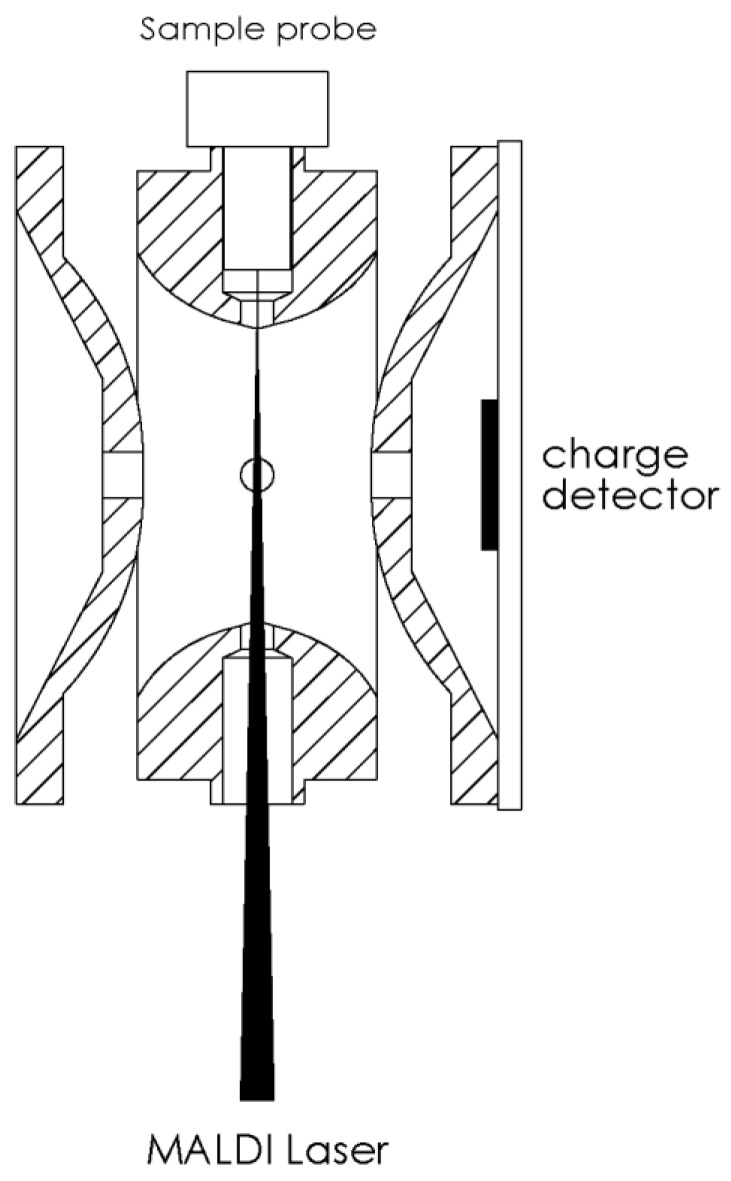
Experimental setup of our homemade MALDI frequency-scanned quadrupole ion trap mass spectrometer with a charge detector.

Mass spectra for biomolecules of *m*/*z* from 10,000 to 1,500,000 were measured by our homemade frequency-scanned QIT mass spectrometer with a charge detector introduced in the previous study [62,68]. The experimental schematic is shown in Figure 8. The sample probe was directly inserted into the center hole on the ring electrode of the ion trap, to avoid any serious ion loss during the transmission from the sample plate to trap. A laser beam (355 nm, Nd: YAG laser, LS-2137U, LOTIS TII, Minsk, Belarus) was focused to a spot, around 0.26 mm in diameter, on the tip of the sample probe in the ion trap. There was no efficiency discrepancy toward the trapping ions with different sizes. A charge detector was mounted on one of the end-caps. There is a circular metal disk at the center of the detector board that was used to act as a Faraday disk to collect the image charges induced by ions. The discharge time constant was determined by the RC value and set as long as 500 µs for detecting large molecular ions.

A lower frequency (2 kHz to 70 kHz) with linear sweeping mode was used for the QIT to trap ions with a large *m*/*z*. For our instrument, the trapping frequency can be changed to cover the different *m*/*z* regions [68]. A broadband power amplifier (PZD2000A, TREK, Inc., Lockport, New York, NY, USA) with output voltages ranging from 0 to ±2000 V direct current (DC) was applied to sustain the radiofrequency (RF) voltage up to 1600 Vpp [56,62] between 2 kHz and 70 kHz, and the stepwise frequency scan [56,62] was used to identify the *m*/*z* values of the ions. The RF voltage was applied to the ring electrode and the two end-caps were both connected to the ground. The QIT was operated in the mass selective axial instability mode [69,70] and was designed to be able to ramp down the trapping frequency at a constant voltage amplitude [71]. For the stepwise scan, the frequency profile is defined by the start frequency (*F*_start_), the frequency increment (∆*f*), and the number of increments (*N*_inc_) per scan. The stepwise scan between the start and end is incrementally increased from *F*_start_ to *F*_start_ + *N*_inc_ × ∆*f*. The ion ejection was completed at a specific frequency before it changed to next frequency. Therefore, the relationship between the ejection frequency and the ion signal is well-defined. The number of ions at every ejecting frequency was measured by the charge detector and the corresponding molecular weight was determined. For the cluster analysis of the biomolecules, each peak of the cluster in the mass spectrum should correspond to a specific frequency, and the signal of the cluster can be acquired by setting the corresponding frequency. The trapping efficiency is related to the depth of the RF potential well, which in turn is related to the *q_z_* value [72]. In order to retain a fairly equal trapping efficiency in the different mass regions, the initial ion-trapping conditions were all fixed at the same *q_z_* value.

Helium was directly injected into the interior part of the ion trap through a tube, and a pressure gauge was installed to get the real helium pressure inside the ion trap. In order to efficiently reduce the kinetic energy of the heavy ions, the helium pressure inside the ion trap was maintained from 50 mTorr to 65 mTorr, so as to provide more collisions. The phase lock between the laser beam and the RF trapping voltage was conducted to select the optimal polarity of ions from MALDI. According to the report published earlier, the phase angle of the positive ions was set as ~90°, to optimize the trapping efficiency [62]. As ion accumulation from multiple laser pulses can lead to a better detection sensitivity [4,56,62], the ions from MALDI were accumulated in the ion trap for all of the experiments. Each mass spectrum was obtained from only one scan, without averaging of more than one spectrum.

**Figure 9:**
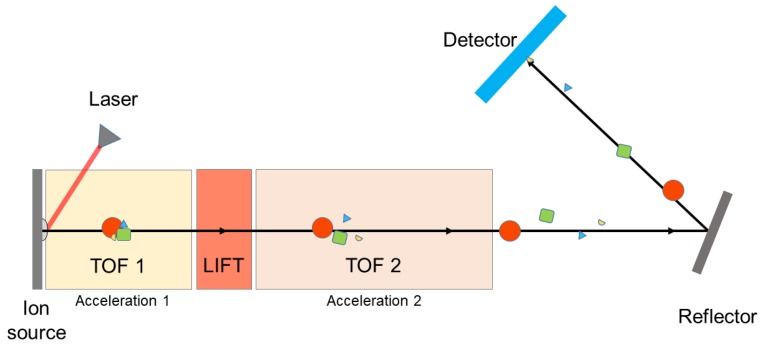
The principle of MALDI-TOF/TOF mass spectrometer.

The cluster spectra of cytochrome c at a lower mass region (10,000–100,000 Da) were measured by a Bruker Ultraflex II MALDI-TOF/TOF mass spectrometer. A schematic for this type of instrument is shown in Figure 9. The masses of the desorbed biomolecular ions are determined by the transit time between desorption and detection. A LIFT (laser-induced fragmentation technology) cell is inserted in the ion flight path, which selects a parent ion and its fragments formed after the source (post-source decay), on the basis of their flight time in the first TOF region. The parent and fragment ions are then re-accelerated, and therefore travel with different velocities according to their masses, and are focused on the detector after passing through the mass reflectron.

### 3.2. Sample Preparation

In the present study, cytochrome c (C2506, Sigma-Aldrich Co., St. Louis, MO, USA) and BSA (A0281, Sigma-Aldrich Co., St. Louis, MO, USA) were employed for obtaining the cluster size distributions. Just like the regular MALDI sample preparation, all of the analyte solutions (100 pmol/μL) were mixed with sinapinic acid (SA) (D7927, Sigma-Aldrich Co., St. Louis, MO, USA) as a matrix. The molecular weight of SA is 224.21. SA was dissolved in a 50:50 water/acetonitrile solution at a concentration of 10 mg/mL. All of the matrix solutions also contained 0.1% trifluoroacetic acid (TFA) (9470-01, Avantor Performance Materials, Inc., Center Valley, PA, USA). To achieve the best ionization efficiency, the molar ratios of the matrix to analyte were 1000 for cytochrome c and 5000 for BSA, according to the results of the preliminary examinations. A single-drop of the sample solution (4 µL per droplet) was placed on the sample probe three times and then air-dried for each experiment.

## 4. Conclusions

In this work, large cluster distributions of cytochrome c and BSA were measured by our homemade frequency-scanned QIT mass spectrometer with a charge detector. The largest cluster ions of these two biomolecules were first observed, which demonstrated the existences of very large bio-clusters in MALDI. To our knowledge, this is the first report for the observation of large biomolecular clusters from the monomer to mega Dalton region. The decreasing tendencies of one and two orders in the relative intensity distributions were observed for cytochrome c and BSA, respectively. The power–law dependence distributions with a very high correlation (*r*^2^ > 0.99) can serve as experimental evidence for the laser ablation studies of MALDI.
